# Screening of Apoptosis Pathway-Mediated Anti-Proliferative Activity of the Phytochemical Compound Furanodienone against Human Non-Small Lung Cancer A-549 Cells

**DOI:** 10.3390/life12010114

**Published:** 2022-01-13

**Authors:** Ahmed Al Saqr, El-Sayed Khafagy, Mohammed F. Aldawsari, Khaled Almansour, Amr S. Abu Lila

**Affiliations:** 1 Department of Pharmaceutics, College of Pharmacy, Prince Sattam Bin Abdulaziz University, Al-Kharj 11942, Saudi Arabia; e.khafagy@psau.edu.sa (E.-S.K.); moh.aldawsari@psau.edu.sa (M.F.A.); 2 Department of Pharmaceutics and Industrial Pharmacy, Faculty of Pharmacy, Suez Canal University, Ismailia 41522, Egypt; 3 Department of Pharmaceutics, College of Pharmacy, University of Hail, Hail 81442, Saudi Arabia; kh.almansour@uoh.edu.sa; 4 Department of Pharmaceutics and Industrial Pharmacy, Faculty of Pharmacy, Zagazig University, Zagazig 44519, Egypt; a.abulila@uoh.edu.sa

**Keywords:** A549 cells, anticancer, apoptosis, cell cycle arrest, non-small cell lung carcinoma, phyto-compound

## Abstract

Furanodienone (FDN), a major bioactive component of sesquiterpenes produced from *Rhizoma Curcumae*, has been repeatedly acknowledged for its intrinsic anticancer efficacy against different types of cancer. In this study, we aimed to investigate the cytotoxic potential of furanodienone against human lung cancer (NSCLC A549) cells in vitro, as well as its underlying molecular mechanisms in the induction of apoptosis. Herein, we found that FDN significantly inhibited the proliferation of A549 cells in a dose-dependent manner. In addition, treatment with FDN potentially triggered apoptosis in A549 cells via not only disrupting the nuclear morphology, but by activating capsase-9 and caspase-3 with concomitant modulation of the pro- and antiapoptotic gene expression as well. Furthermore, FDN revealed its competence in inducing cell cycle arrest at G0/G1 phase in A549 cells, which was associated with decreased expression of cyclin D1 and cyclin-dependent kinase 4 (CDK4), along with increased expression of CDK inhibitor p21Cip1. Intriguingly, FDN treatment efficiently downregulated the Wnt signaling pathway, which was correlated with increased apoptosis, as well as cell cycle arrest, in A549 cells. Collectively, FDN might represent a promising adjuvant therapy for the management of lung cancer.

## 1. Introduction

Lung cancer is considered the leading cause of cancer-associated mortalities accounting for 1.80 million during 2020. Furthermore, a total of 2,206,771 new cases of lung cancer were reported during the previous year, which constituted 11.4% of all the reported cancer cases. The escalation in the incidence of lung carcinomas makes it a challenging health concern globally.

Lung carcinomas fall categorically either under non-small cell lung cancer (NSCLC), which is responsible for 85% of lung-associated malignancies, or small cell lung carcinomas (SCLC), accounting for the remaining 15% of lung cancer [1]. Clinical management of patients diagnosed with NSCLC initially depends on the diagnosed stage and is subsequently treated using surgical resection, followed by chemo- and/or radiotherapeutics [2]. Nevertheless, although the past few years have witnessed advances in the therapeutic approaches of lung cancer, the overall five-year survival rate is still only 19% [3]. Such poor prognosis in lung cancer patients has been attributed mainly to the development of resistance towards antineoplastic agents [4]. Consequently, there is an urgent need for the development of new anticancer entities that could circumvent the abovementioned problems, along with improving the prognosis for lung cancer patients.

**Scheme 1:**
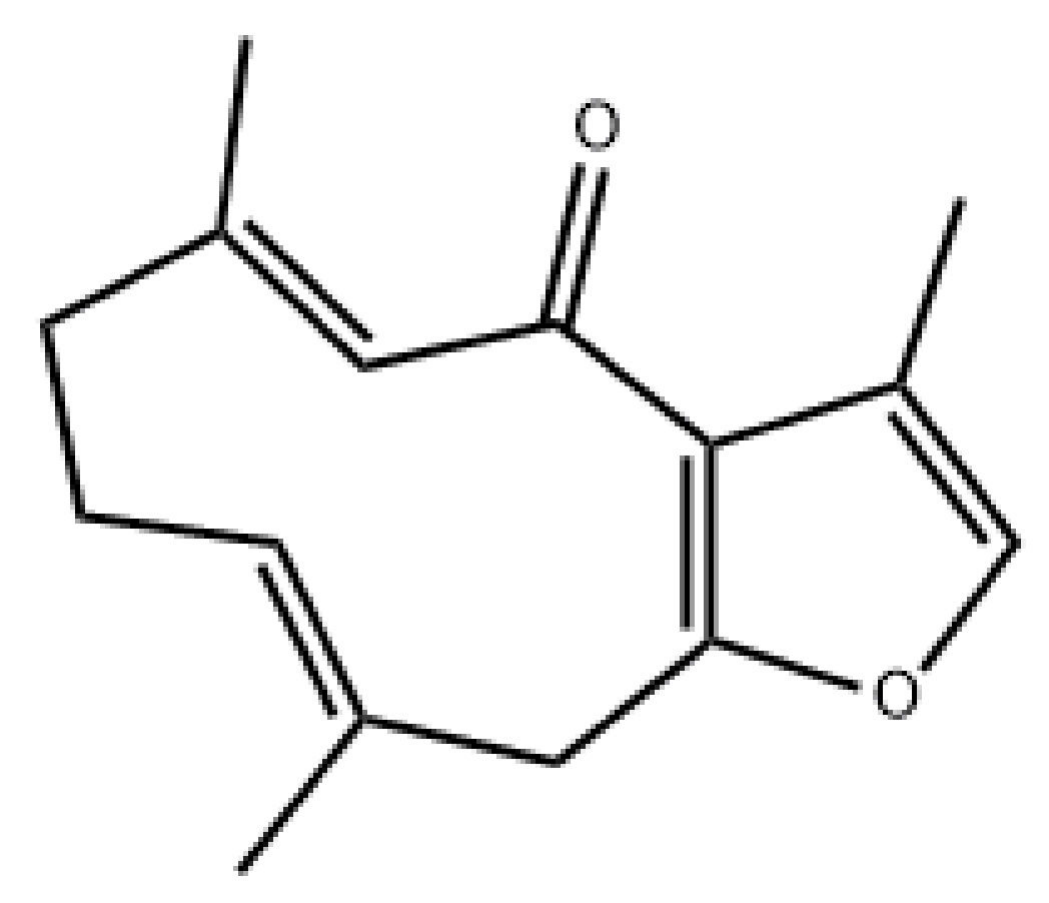
Chemical structure of Furanodienone.

Recently, naturally occurring compounds derived from a plant source, known as phytochemicals, have received increased interest in oncology. They serve as vital resources for novel drugs that might act as natural-based alternatives with less toxicity than conventional chemotherapeutic agents. Furanodienone (FDN) is a furanosesquiterpenoid belonging to furanogermacranes that is commonly extracted from various Curcuma and Commiphora species (Scheme 1). FDN was documented to possess interesting pharmacological activities, such as anti-inflammatory [5] and antimicrobial activities [6]. Most importantly, FDN has been recently reported to exert potent cytotoxic activity against different cancer cell lines, including colorectal cancer and breast cancer, through the induction of apoptosis [7,8]. However, the cytotoxic potential of FDN against lung cancer cells along with the possible molecular mechanisms of its inhibitory activity have not been investigated yet.

In cancer therapy, the anticancer effect of phytochemicals, including FDN, often act via regulating molecular pathways involved in cancer growth and progression. For instance, FDN was reported to trigger G0/G1 arrest and to induce apoptosis in human colorectal cancer cells via reactive oxygen species/mitogen-activated protein kinases (ROS/MAPKs)-mediated caspase-dependent pathway [7]. Furthermore, FDN induced cell cycle arrest and apoptosis in human epidermal growth factor receptor 2 (HER2)-overexpressing human breast cancer cells via interfering with EGFR/HER2 signaling [8]. The aim of the current study, therefore, was to investigate the antiproliferative effects and molecular mechanisms of furanodienone on A549 lung cancer cells in vitro. The results of our study revealed for the first time the evidence that FDN causes G0/G1 phase arrest and induces apoptosis via instigating mitochondria-mediated apoptosis in A549 cells, through interfering with the Wnt signaling pathway.

## 2. Materials and Methods

### 2.1. Materials

Furanodienone was obtained commercially from MedChemExpress, Monmouth Junction, NJ, USA (Cas No. 24268-41-5). Cisplatin, MTT ((3-(4,5-dimethylthiazol-2-yl)-2,5-diphenyltetrazolium bromide), rhodamine-123 (Rh-123), N-acetyl cysteine (NAC), 2,7-dichlorodihydrofluorescein diacetate (DCFH-DA), Hoechst-33342, caspase-9 (APT173), and caspase-3 (CASP3C-1KT) kits, along with respective inhibitors, were obtained from Sigma Aldrich (St. Louis, MO, USA). Kaighn′s modification of Ham′s F-12 (F-12K) medium, fetal bovine serum (FBS), and antibiotic–antimycotic solution used in the study were from Gibco (Fort Worth, TX, USA). FITC Annexin-V apoptosis assay kit was from BD Bioscience (San Diego, CA, USA). Primers used in the study were obtained from Integrated DNA Technologies (IDT; Coralville, IA, USA).

### 2.2. Cell Line

Human NSCLC A549 cells and murine alveolar macrophages J774A.1 were obtained from the American Type Culture Collection. Human NSCLC A549 cells were cultured in Kaighn′s modification of Ham′s F-12 medium (F-12K), while J774A.1 were cultured in Dulbecco′s modified Eagle′s medium (DMEM). Both media were supplemented with FBS (10% *v*/*v*) and an antibiotic–antimycotic cocktail (1% *v*/*v*). During the experimental procedures, the cells were maintained in ambient culture conditions comprising the humidified atmosphere at 37 °C with 5% CO_2_. A predetermined number of cells were used specifically for every individual experiment post-quantification of viable cell numbers through 0.4% trypan blue solution aided by hemocytometer.

### 2.3. Cytotoxicity Estimation

The cytotoxicity of furanodienone (FDN) on A549 cells was initially evaluated at varying concentrations, ranging from 0 to 300 µM, to determine the appropriate FDN concentration(s) to be used throughout the study using the standard procedure of MTT as previously described [9]. From this study, FDN concentrations of 50, 100, and 200 μM, which exerted remarkable cytotoxic potential, were selected to be used throughout the whole study.

Next, cell viability of either NSCLC A549 or murine alveolar macrophage J774A.1 cells was evaluated upon treatment with different FDN concentrations (50, 100, and 200 μM). For this purpose, A549 or J774A.1 cells at a cell density of 5 × 10^3^ cells/well were exposed to FDN for 24 h. Wells containing untreated cells served as the negative control, while wells containing cells treated with a standard cytotoxic agent (cisplatin; 200 μM) served as the positive control. At 24 h post-incubation, the wells were decanted and 10 µL MTT dye from a stock of 5 mg/mL was added to each well, followed by an incubation of 4 h in ambient culture conditions as stated above. The wells were subsequently supplemented with dimethyl sulfoxide (DMSO; 0.1 mL) and the plate was vortexed gently for 30 min in the dark before recording the absorbance of each well at a wavelength of 490 nm using Bio-Rad spectrophotometer (Hercules, CA, USA). The results were expressed as cell viability (%), determined using the formula A_assay_/A_control_ × 100 (A_assay_: absorbance of treated groups and Ac_ontrol_: absorbance of the control group).

### 2.4. Effects on Morphological Attributes

The morphologies of untreated control and FDN-exposed A549 cells were comparatively assessed. Briefly, 1 × 10^4^ A549 cells/well were exposed to the stated concentrations of FDN and were incubated for 24 h. Subsequently, cells belonging to each treated group were visualized for alterations in their morphology through FLoid Imaging Station, Thermo-Scientific (Waltham, MA, USA).

### 2.5. Alterations in Nuclear Morphology

The initiation of apoptosis within A549 cells subjected to the stated FDN concentrations was assessed through evaluating the modifications within nuclear morphology by Hoechst-33342 stain as described previously [10]. A549 cells (5 × 10^3^) were exposed to varying concentrations (50, 100, and 200 µM) of FDN and were maintained for at least 24 h under ambient culture conditions. Untreated cells served as controls. Subsequently, after removing the spent media, cells were re-exposed with 5 µg/mL of Hoechst-33342 and incubated shortly for 10 min in ambient culture conditions. The cells were then visualized for their fluorescence through the blue filter of FLoid Imaging Station (Thermo-Scientific, Waltham, MA, USA). For quantification, the fluorescence intensities of the stained cells were detected by image J program (NIH, LOCI, University of Wisconsin, Madison, WI, United States) [11]. The mean signal intensities and standard deviations were calculated for at least 25 cells per each preparation and control. One-way ANOVA test followed by Tukey′s multiple comparison post-test were used to attest the statistical significance, where *p* < 0.05 was considered significant.

### 2.6. Annexin V/FITC Assay

The efficacy of FDN in instigating apoptosis post-treatment in A549 cells was quantified using Annexin V-FITC/PI apoptosis kit (BD Biosciences, San Diego, CA, USA). Briefly, 4 × 10^5^ cells were seeded in each well of a six-well plate and allowed to adhere overnight. Thereafter, the cells were exposed to varying FDN concentrations (50, 100, and 200 µM) for 24 h. At 24 h post-incubation, the cells in each well were washed trice prior to the addition of 1× binding buffer as per the instructions supplied by the manufacturer. Eventually, nearly 1 × 10^5^ cells constituting to approximately 100-110 µL suspension was treated with 5 µL each of Annexin V-FITC and PI, followed by a brief incubation (15 min) in the dark at 37 °C. Thereafter, the volume of the cell suspension was increased up to 500 µL by adding binding buffer (1×). The suspension was then evaluated using flow cytometer FACS Aria (BD Biosciences, San Diego, CA, USA).

### 2.7. Caspase Assay

Colorimetric assay kits specific for caspase-9 and -3 were used to estimate the caspase activities in human lung cancer cells. After incubation with the above stated FDN concentrations, approximately 3 × 10^6^ A549 cells were ruptured through 50 µL of ice-cold lysis buffer following 10 min incubation on ice. The cellular lysate was thereafter centrifuged for 1 min at 10,000× *g* and 4 °C. Subsequently, 50µL of lysate was added in each of a 96-well plate with equal volumes of 10 mM dithiothreitol (DTT) constituted reaction buffer. Thereafter, 4 mM substrate (DEVD-pNA) was supplemented per well and allowed to incubate for 10 min. Post-incubation, the plate was read for the absorbance at 405 nm. The results were interpreted as percentage (%) increase in the activity of specific caspases by comparing the absorbance with the untreated control.

To assess the effect of pretreatment of caspase inhibitors on cell viability, A549 lung cancer cells were pretreated with caspase-9 (Z-LEHD-FMK) and caspase-3 (Z-DEVD-FMK) inhibitors (50 µM) for 2 h. The cell viability was then estimated by MTT assay after 24 h of FDN treatment.

### 2.8. Assessment of FDN-Induced Alterations in ROS

The efficacy of FDN in instigating levels of ROS within NSCLC A549 cells was assessed through DCFH-DA stain as per the protocol described earlier [12]. For qualitative assessment, 2 × 10^4^ A549 cells/well were treated with 50, 100, and 200 µM of FDN and, at 12 h post-incubation, cells were stained by DCFH-DA (10 µM; 30 min at room temperature). Finally, the cells were visualized and the photomicrographs were captured through the green filter of FLoid Imaging Station (Thermo-Scientific, Waltham, MA, USA).

For quantification of ROS generated post-FDN treatment, 2 × 10^4^ A549 cells were seeded in a black-bottom 96-well plate and subsequently exposed to FDN (50, 100, and 200 µM) for 12 h under optimum culture conditions. Post-FDN exposure, cells were retreated with 10 µM DCFH-DA at room temperature for 30 min and recorded for their DCF-DA-instigated fluorescence intensity through Synergy H1 Hybrid Reader (BioTek, Winooski, VT, USA). The results were elucidated as percentage of mean DCF-DA intensity compared with the untreated control.

### 2.9. Alterations within Mitochondrial Membrane Potential (ΔΨm)

The potential of FDN to alter mitochondrial membrane potential (ΔΨm) within treated A549 cells was investigated using mitochondria voltage-specific Rh-123 dye as described earlier [13]. Briefly, 1 × 10^5^ A549 cells were treated with 50, 100, and 200 µM FDN and incubated overnight under ambient culture conditions. Subsequently, the cells were stained using 5 mg/mL of Rh-123 for 30 min. Finally, the fluorescence associated with cells were visualized and recorded through FLoid Imaging Station (Thermo-Scientific, Waltham, MA, USA).

Furthermore, MMP was quantitatively estimated by using Rh123 staining. Briefly, A549 cells were seeded in a 96-well plate, followed by the treatment with different doses of FDN. Thereafter, the plate was aspirated and the A549 cells were incubated with Rh123 for 30 min. Fluorescence was analyzed at an excitation wavelength of 488 nm and an emission wavelength of 525 nm.

### 2.10. Instigation of Cell Cycle Arrest

The efficacy of FDN in instigating cell cycle arrest within A49 cells was assessed using propidium iodide (PI) stain as described previously [14]. Briefly, 5 × 10^5^ A549 cells were incubated for 24 h under standard culture conditions post-FDN treatment. Subsequently, cells from each group were centrifuged (1200 rpm, 10 min, 37 °C) and the pellet was treated with RNAse-A (50 µg/mL, 30 min) at room temperature. The cells were then fixed through 12 h incubation at −20 °C with chilled ethanol. Eventually, the cells were stained with 25 µg/mL of PI (15 min) at 37 °C and analyzed using FACS ARIA (BD Biosciences, San Diego, CA, USA).

### 2.11. mRNA Expression (Quantitative RT-PCR)

A total of 1 × 10^6^ A549 cells were treated with different concentrations of FDN (50, 100, and 200 µM) and were incubated under optimum culture conditions. Untreated cells served as controls. At 24 h post-incubation, RNA from different groups was separated using a commercial kit from Thermo Scientific (Waltham, MA, USA). cDNA was subsequently synthesized from 2 µg of isolated RNA through a commercially procured kit. Primers used for synthesis of cDNA were described previously and are stated in Table 1 [14,15,16,17]. Afterwards, quantitative RT-PCR was performed through a commercially available qPCR kit (Thermo Scientific, Waltham, MA, USA), during which glyceraldehyde 3-phosphate dehydrogenase (*GAPDH*) gene was used for normalization. Relative CT methodology was used to quantify the alterations within expression of different genes in comparison with *GAPDH* gene, which, in turn, was set to be 1. The comparative levels of mRNA for an individual gene were normalized to the mean of the respective control and were estimated by ΔΔCT method.

### 2.12. Statistical Analysis

The reported data represent mean ± SD of three independent experiments replicated in triplicate. Statistical significance was quantified through GraphPad Prism Ver.5.0 (San Diego, CA, USA) and was deemed to be significant when *p* < 0.05 using one-way ANOVA, subsequently followed by Dunnett and paired Student t-test post hoc test. * *p* < 0.05, ** *p* < 0.01, and *** *p* < 0.001.

## 3. Results

### 3.1. FDN Inhibited the Growth of A549 Cells

**Figure 1:**
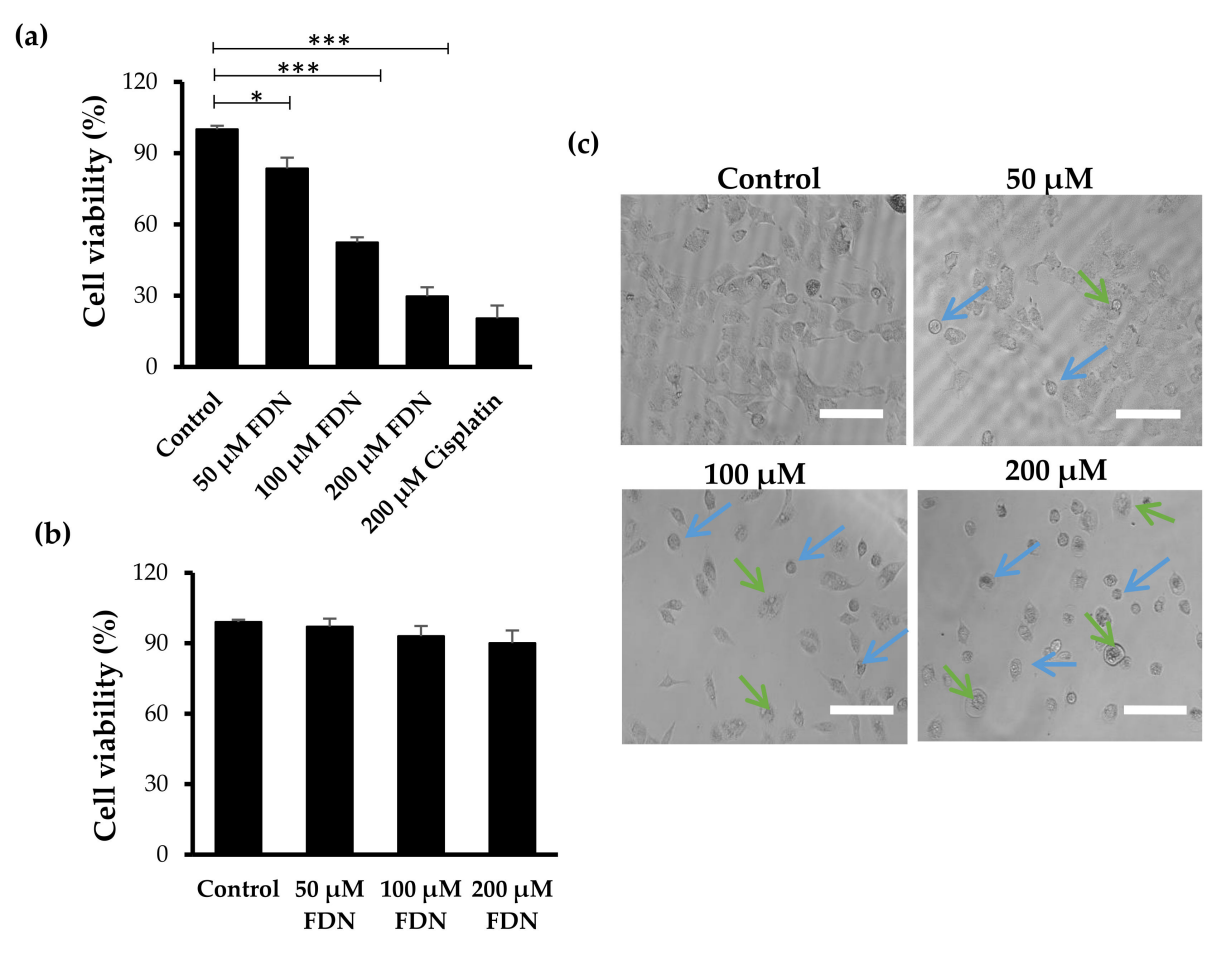
FDN mediated toxic effects on (**a**) NSCLC A549 cells and (**b**) J774A.1 cells as established through MTT assay. (**c**) Phase-contrast images of FDN-treated A549 cells for 24 h, instigating morphological aberrations where swelling and cell membrane lysis within FDN-treated A549 cells are indicated by green and blue arrows (scale bar 100 µm). * p < 0.05, *** p < 0.001 vs. control.

A cell survival assay was adopted to investigate the anticancer efficacy of FDN against A549 cells. Initially, A549 cells were treated with various concentrations of FDN ranging from 0 to 300 μM. As shown in Figure S1, FDN induced a dose-dependent cytotoxic effect against A549 cells, with a calculated IC_50_ value of 85.02 μM. According to the cell viability curve (Figure S1), FDN concentrations of 50, 100, and 200 μM, which exerted remarkable cytotoxic potential, were selected to be used throughout the whole study. In the cell viability study (Figure 1a), FDN efficiently inhibited the growth of A549 cells, in a dose-dependent manner, implicating the inhibitory effects of FDN. FDN reduced the viability of A549 cells to 83.46 ± 4.61%, 52.40 ± 2.17%, and 29.73 ± 3.81% upon treatment with 50, 100, and 200 µM FDN, respectively. Of interest, the cytotoxic potential of 200 μM FDN was comparable to that of a standard compound, cisplatin. In addition, the cytotoxic potential of FDN was investigated against noncancerous cells via MTT assay. As shown in Figure 1b, an insignificant decrease in the viability of J774A.1 cells was observed upon treatment with various concentrations of FDN for 24 h, as compared to the control untreated cells. These results emphasized the cytotoxic potential of FDN against cancerous A549 NSCLC but not normal J774A.1 cells. In addition, morphological analysis (Figure 1c) by phase contrast microscopy revealed that treatment of A549 cell line with FDN for 24 h resulted in low cell confluence and plasma membrane blebbings, suggesting the induction of cell apoptosis. Furthermore, FDN treatment reduced the adherence of A549 cells, resulting in floating of cells, which was not obvious with untreated control cells that remained adhered under the same culturing conditions.

### 3.2. FDN Induced Apoptosis via Intrinsic Apoptosis Pathway

**Figure 2:**
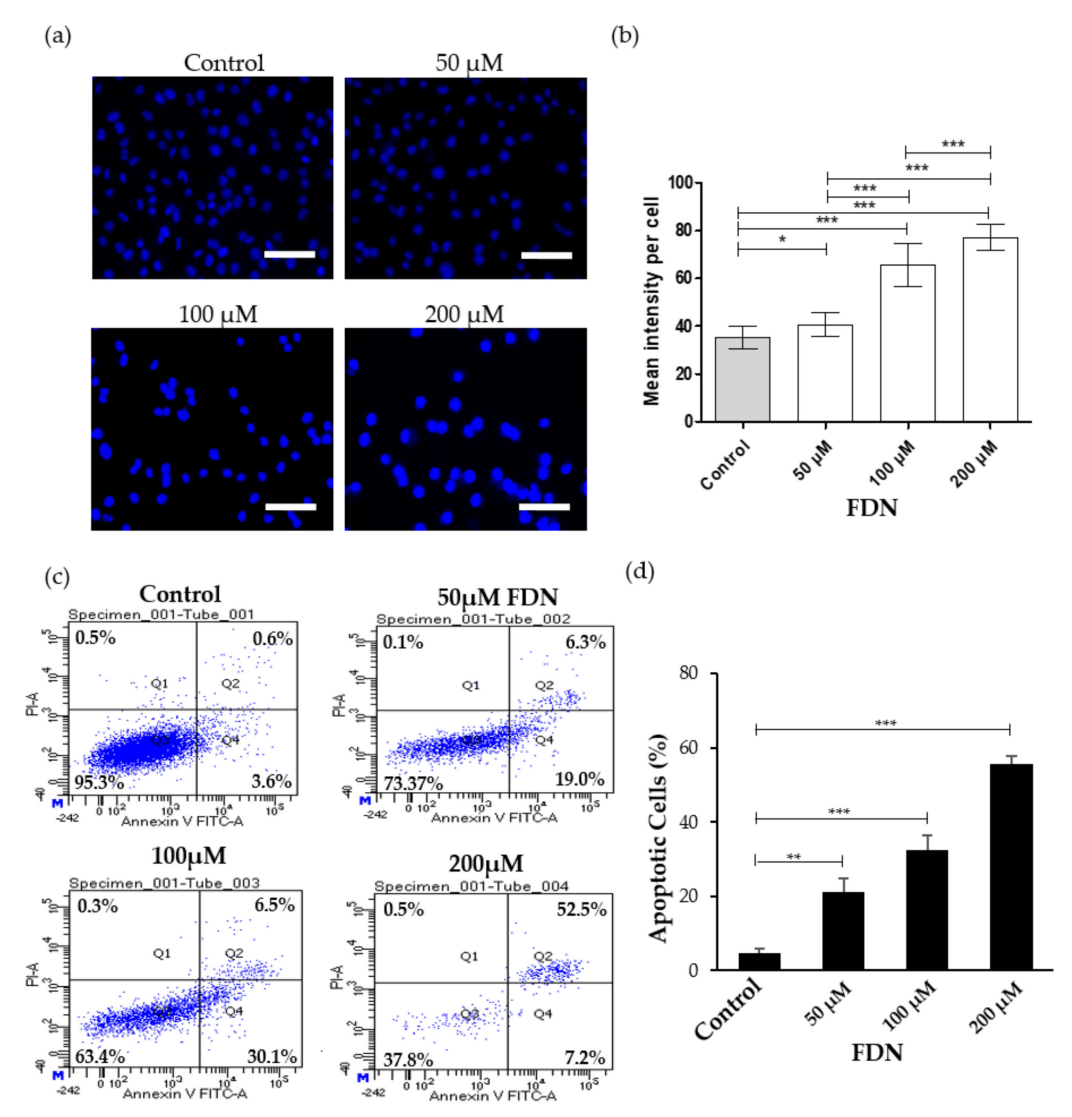
(**a**) Condensation and fragmentation of nucleus post-FDN exposure (scale bar 100 µm); (**b**) signal intensity of Hoechst-33342 staining. The mean signal intensities were determined for at least 25 cells from each preparation and control using image J program. (**c**) FDN induced apoptosis in lung cancer cells as assessed by Annexin-V FITC/PI assay. (**d**) Percent total apoptosis in FDN-treated lung cancer cells normalized to the number of untreated cells. * p < 0.05, ** p < 0.01, *** p < 0.001 vs. control.

To examine whether FDN was able to induce apoptosis in A549 lung cancer cells, Hoechst-33342 staining was conducted [18]. As shown in Figure 2a, FDN-treated cells, stained with Hoechst-33342, exhibited brighter blue fluorescence compared to untreated control cells, particularly at higher FDN concentrations, indicating higher chromatin condensation. These results suggest that FDN exerted its cytotoxic potential, at least in part, via the induction of apoptosis in lung cancer cells. Quantification of fluorescence intensity supports our findings, where higher fluorescence intensity was observed in FDN-treated cells compared to control cells (Figure 2b).

In addition, apoptosis was also quantitatively estimated by Annexin V-FITC/PI assay to estimate the efficacy of FDN in inducing apoptosis in A549 cells. As observed in Figure 2c, cells in the upper right quadrant (UR) undergo early-stage apoptosis (Annexin V-FITC^+^, PI^−^), and cells in the lower right quadrant (LR) witnessing late-stage apoptosis (Annexin V-FITC^+^, PI^+^) were referred to as apoptotic cells. The sum of LR and UR is reported as total apoptosis, which, in turn, was normalized with the apoptotic cells in the untreated control (4.7 ± 1.03%). The percentage of A549 cells that had undergone apoptosis was increased up to 21.08 ± 3.70%, 32.43 ± 4.06%, and 55.57 ± 2.07% when treated with 50 µM, 100 µM, and 200 µM FDN, respectively (Figure 2d). A minimum of 10,000 events were recorded for each Annexin V-FITC analysis.

### 3.3. FDN Instigated Caspase Apoptotic Pathways in A549 NSCLC Cells

**Figure 3:**
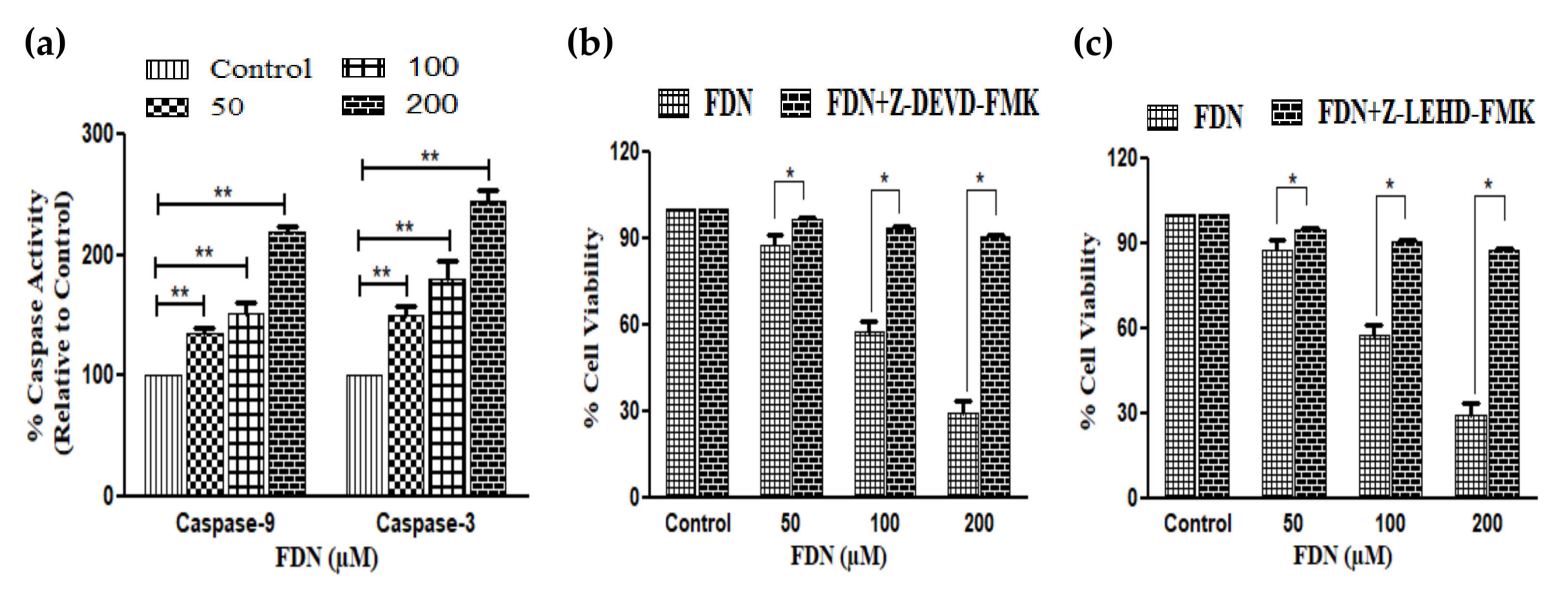
(**a**) Effect of FDN on caspase-3 and -9 activities. Cellular viability of NSCLC A549 cells pre-exposed to inhibitors of (**b**) caspase-3 and (**c**) caspase-9. * p < 0.05, ** p < 0.01 vs. control.

In order to elucidate the cellular events associated with apoptosis induction in A549 lung cancer cells upon treatment with FDN, caspase assay was conducted. As shown in Figure 3a, treatment with FDN substantially enhanced caspase-9 and caspase-3 activities in a dose-dependent manner. Treatment with 200μM FDN significantly enhanced caspase-9 and caspase-3 activities by 118.58 ± 3.66% and 144.54 ± 6.44%, respectively, compared to treatment with 50 μM FDN (35.98 ± 5.41% and 49.69 ± 6.63%, respectively).

Furthermore, to delineate the contribution of caspases in FDN-mediated apoptosis in lung cancer, A549 cells were pretreated for 2 h with Z-DEVD-FMK (caspase-3 inhibitor) and Z-LEHD-FMK (caspase-9 inhibitor) prior to treatment with FDN (50–200μM) for 24 h. As shown in Figure 3b,c, pretreatment with caspase inhibitors entirely abrogated FDN-mediated apoptosis in A549 cells. These results strongly confirmed the pivotal role of caspase activation in FDN-mediated apoptosis in A549 lung cancer cells.

**Figure 4:**
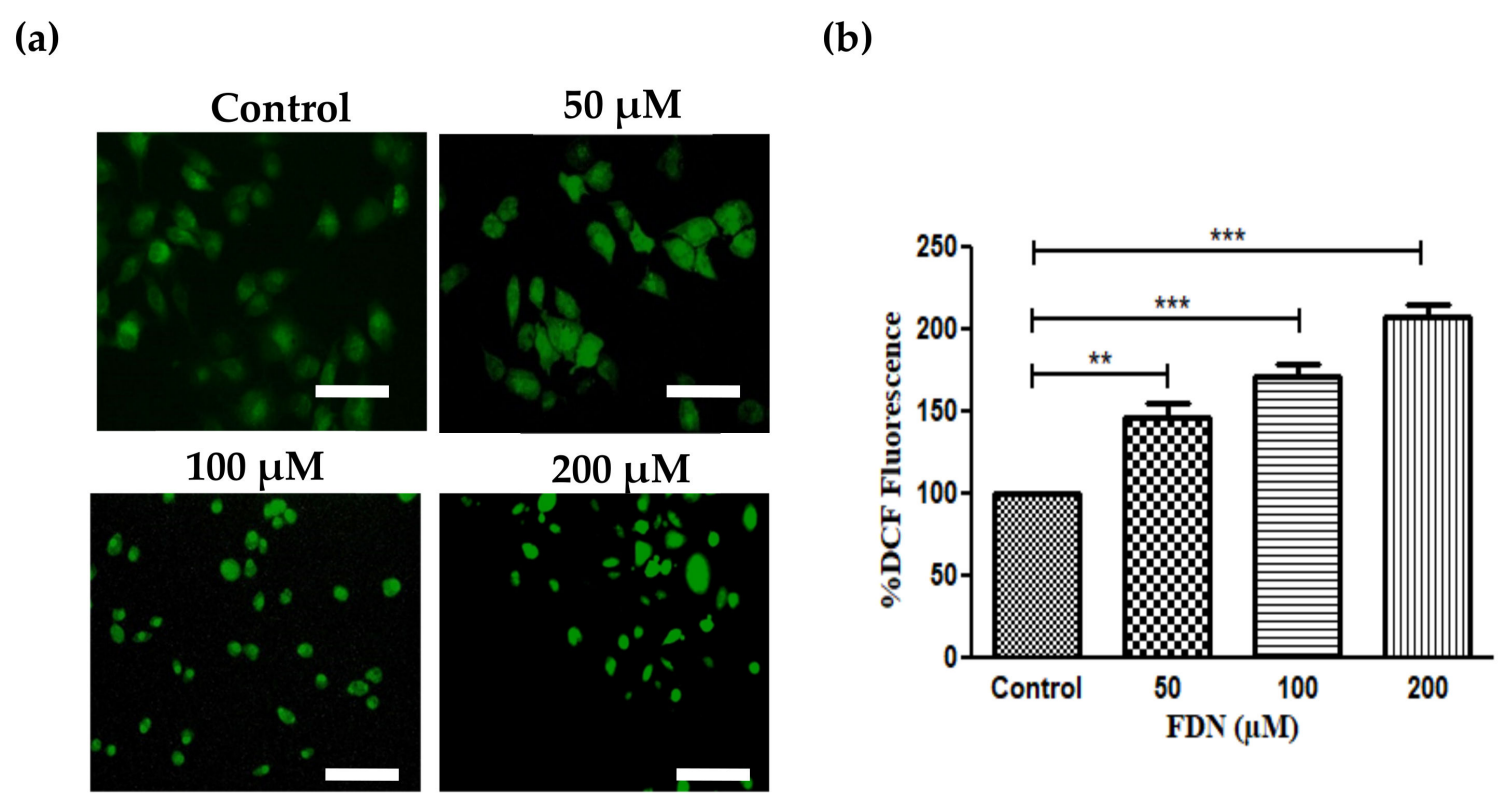
Intracellular ROS generation post-FDN exposure assessed (**a**) qualitatively (scale bar 100 µm) and (**b**) quantitatively using DCFH-DA staining. ** p < 0.01, *** p < 0.001 vs. control.

Reactive oxygen species (ROS) production is considered one of the driving forces in the instigation of apoptotic pathways [14]. Accordingly, levels of ROS were assessed in FDN-treated and untreated A549 cells. As depicted in Figure 4a, FDN-treated cells exhibited substantially stronger DCF-DA-mediated fluorescence compared to untreated control cells. These results obviously indicated the efficiency of FDN in augmenting ROS production within A549 cells. Similarly, quantification of DCF-DA-mediated fluorescence intensity revealed that the percentage DCF-DA fluorescence was 146.32 ± 0.08% at 50 µM FDN, 164.62 ± 0.06% at 100 µM FDN, and 201.46 ± 0.02% at 200 µM FDN, compared with untreated cells (Figure 4b). This evidence clearly revealed that FDN incited ROS generation within A549 cells in a dose-dependent manner.

### 3.4. FDN Triggered Mitochondrial-Mediated Apoptosis

**Figure 5:**
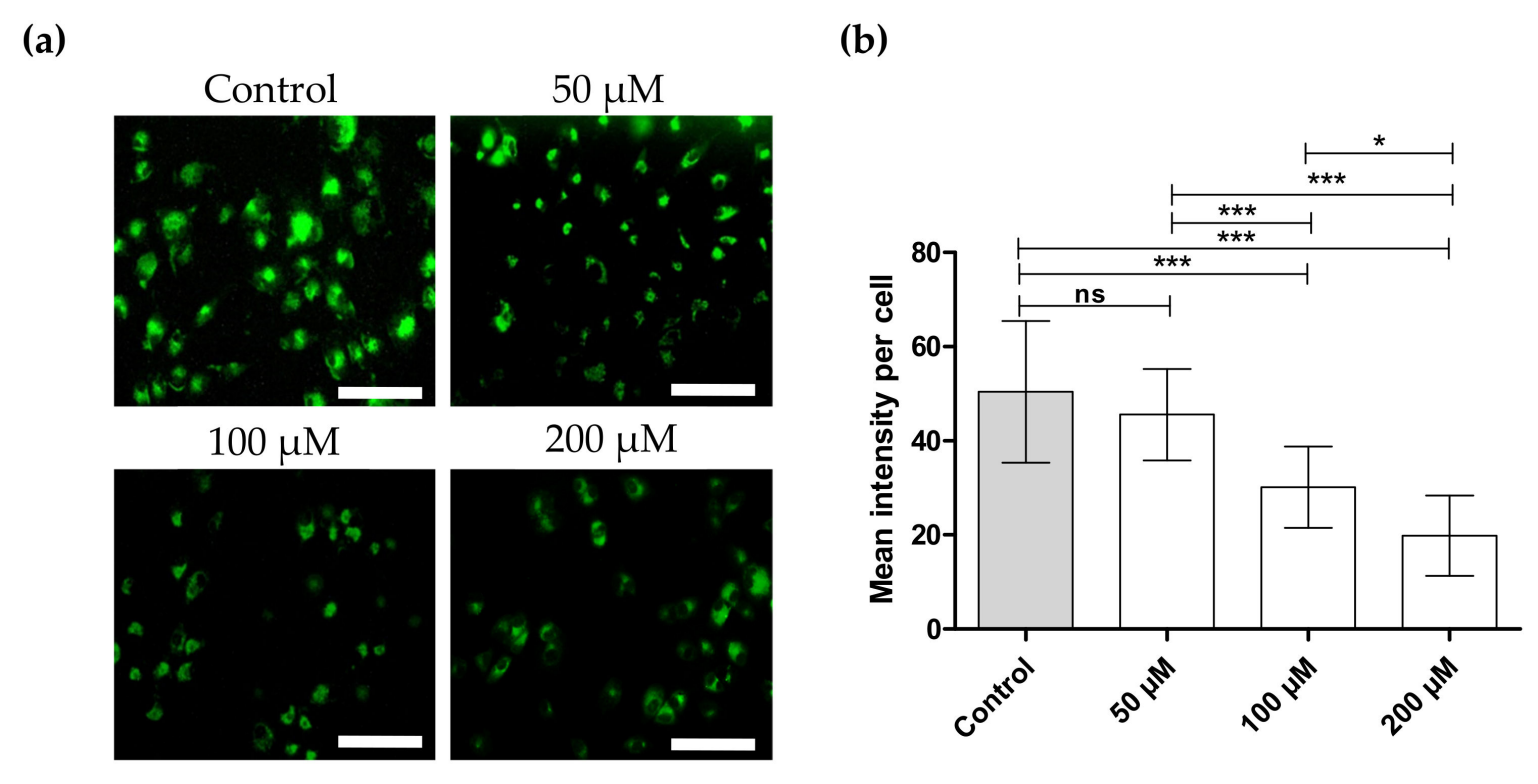
(**a**) Dissipation of Δψm post-FDN treatment at respective dosage (scale bar 100 µm); (**b**) mean signal intensities were determined for at least 25 cells from each preparation and control using image J program. * p < 0.05, *** p < 0.001 vs. control.

The interruption of normal mitochondrial function, particularly alterations that affect the mitochondrial membrane potential (ΔΨm), is a distinguishing feature of apoptosis [19]. Accordingly, alteration in ΔΨm was assessed in A549 lung cancer cells following treatment with FDN. As depicted in Figure 5, in the absence of FDN, A549 lung cancer cells showed an intact ΔΨm, manifested by the cellular uptake of rhodamine 123 dye and high fluorescence intensity. On the other hand, upon treatment with FDN (50, 100, and 200μM), a remarkable reduction in rhodamine 123 dye uptake by A549 cells was observed, as manifested by a dose-dependent reduction in fluorescence intensity, due to loss of ΔΨm. These results suggest that FDN triggered a remarkable drop in ΔΨm, which, in turn, might contribute to the apoptotic potential of FDN against A549 lung cancer cells.

Furthermore, to quantify the altered ΔΨm in A549 lung cancer cells upon treatment with FDN, fluorescence intensities of Rh123 dye were quantified. As shown in Figure 5b, FDN treatment decreased the fluorescence intensity in a dose-dependent manner in A549 lung cancer cells. The fluorescence intensity in cells treated with 200 μM FDN was significantly lower than that in the control group. 

**Figure 6:**
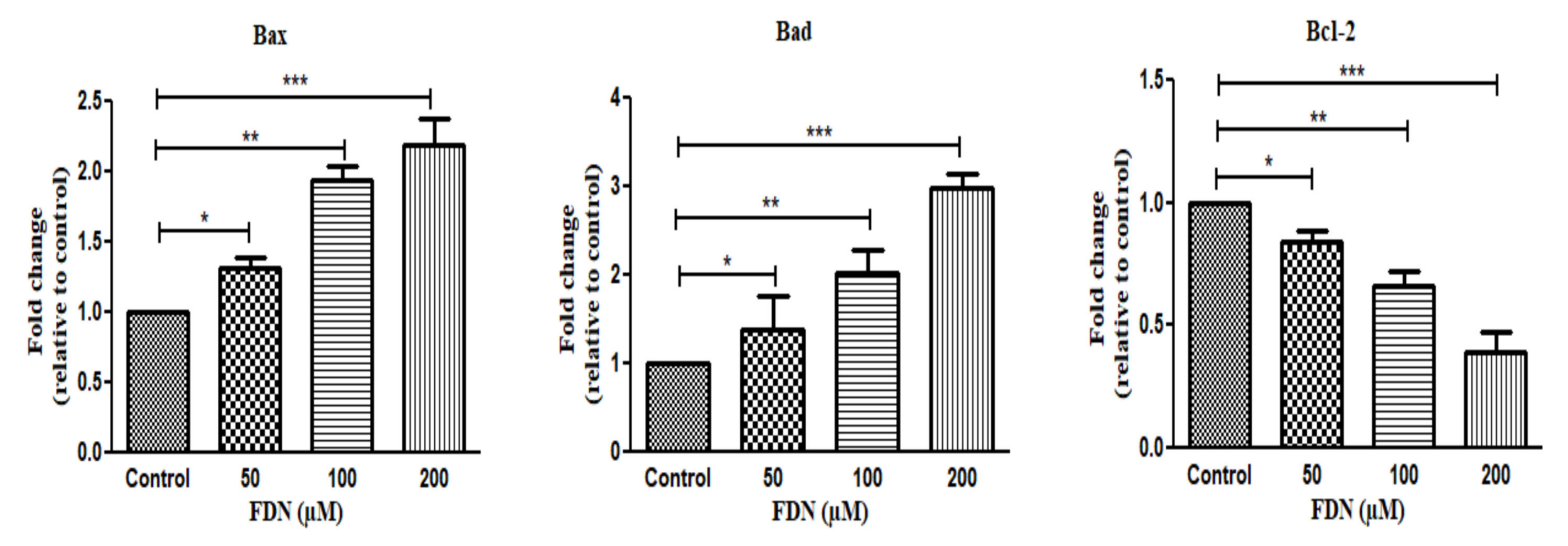
Competency of FDN in inducing the alterations within expression of pro- and antiapoptotic genes as evaluated through RT-PCR. * p < 0.05, ** p < 0.01, *** p < 0.001 vs. control.

In order to further verify the contribution of mitochondria to FDN-induced apoptosis in A549 lung cancer cells, the mRNA expression levels of some Bcl-2 family members, namely Bax, Bad (pro-apoptotic protein), and Bcl-2 (anti-apoptotic), which are crucial mediators of the mitochondrial apoptotic pathway, were evaluated using qRT-PCR analysis. As shown in Figure 6, treatment with FDN significantly enhanced the expression levels of proapoptotic proteins (Bax and Bad) in a dose-dependent manner. Treatment with 50, 100, and 200μm FDN resulted in a 1.31-, 1.94-, and 2.19- and 1.38-, 2.02-, and 2.98-fold change in the expression levels of both Bax and Bad, respectively, when compared with untreated cells (Figure 6). Simultaneously, FDN efficiently alleviated the expression levels of the antiapoptotic protein Bcl-2 compared to control cells. These results collectively indicated that FDN treatment succeeded in elevating the expression levels of proapoptotic proteins, with mutual reduction in the expression of antiapoptotic proteins in A549 cells.

### 3.5. FDN Impeded the Progression of A549 Cells at G0/G1 Phase

**Figure 7:**
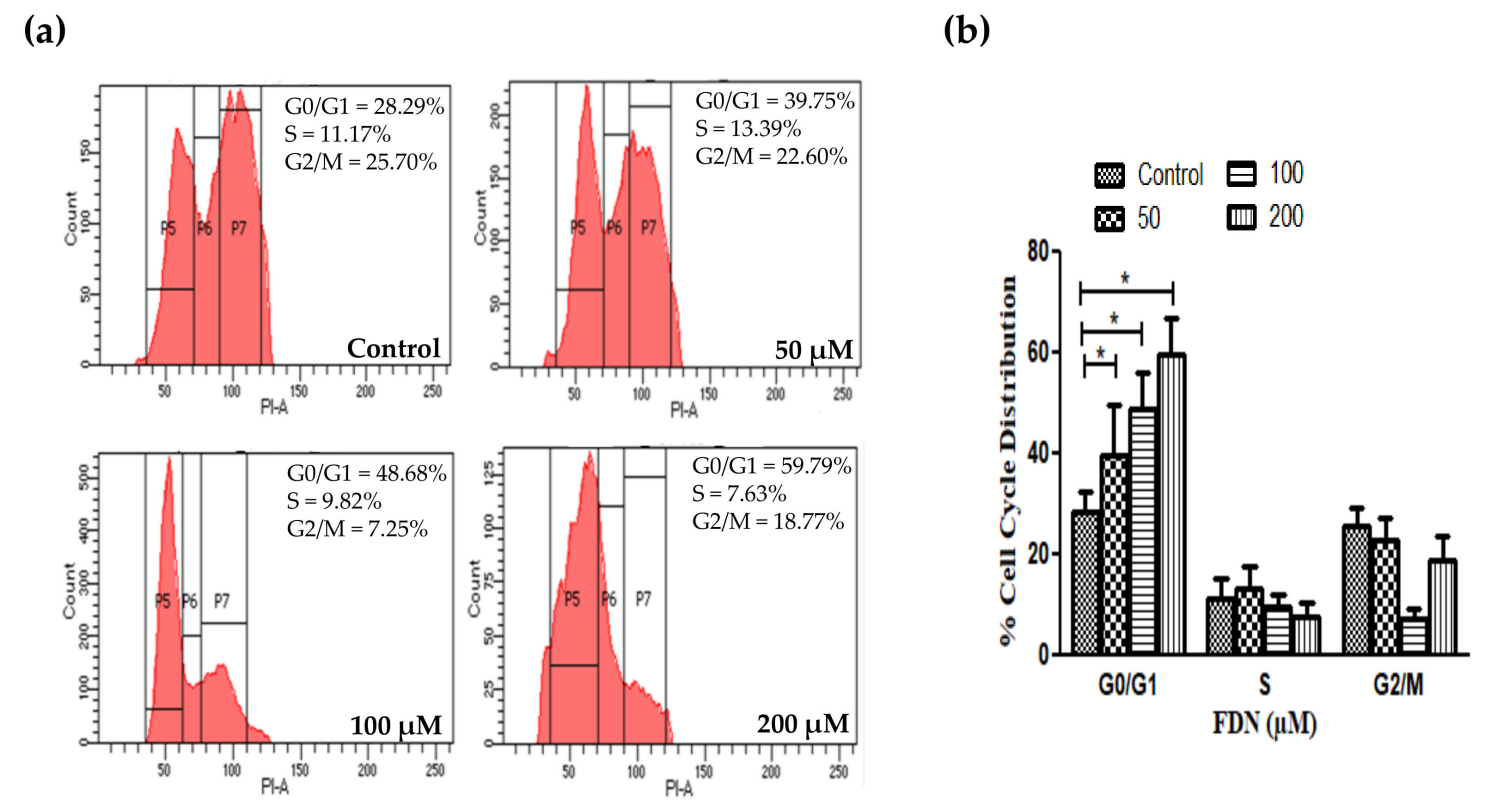
The effect of FDN treatment on cell cycle progression of A549 lung cancer cells. (**a**) Cell cycle analysis by flow cytometry of A549 cells treated with FDN (50-200 μM) for 24 h. (P5 = G0/G1, P6 = S and P7 = G2/M). (**b**) Quantitative data from three independent experiments. * *p* < 0.05 vs. control.

In order to examine whether alterations in the cell cycle distribution were responsible for A549-mediated cell growth inhibition and apoptosis induction, the population of A549 cells in different cell cycle phases was quantified by flow cytometry following treatment with FDN. Treatment of A549 cells with 50, 100, or 200μm FDN for 24 h led to a dose-dependent accumulation of cells at G0/G1 phase compared to untreated cells. The G0/G1 percentages were 39.75 ± 5.28% (50 µM), 48.68 ± 5.85% (100 µM), and 59.79 ± 5.83% (200 µM), respectively, compared to untreated cells (28.29 ± 3.28%) (Figure 7a,b). 

**Figure 8:**
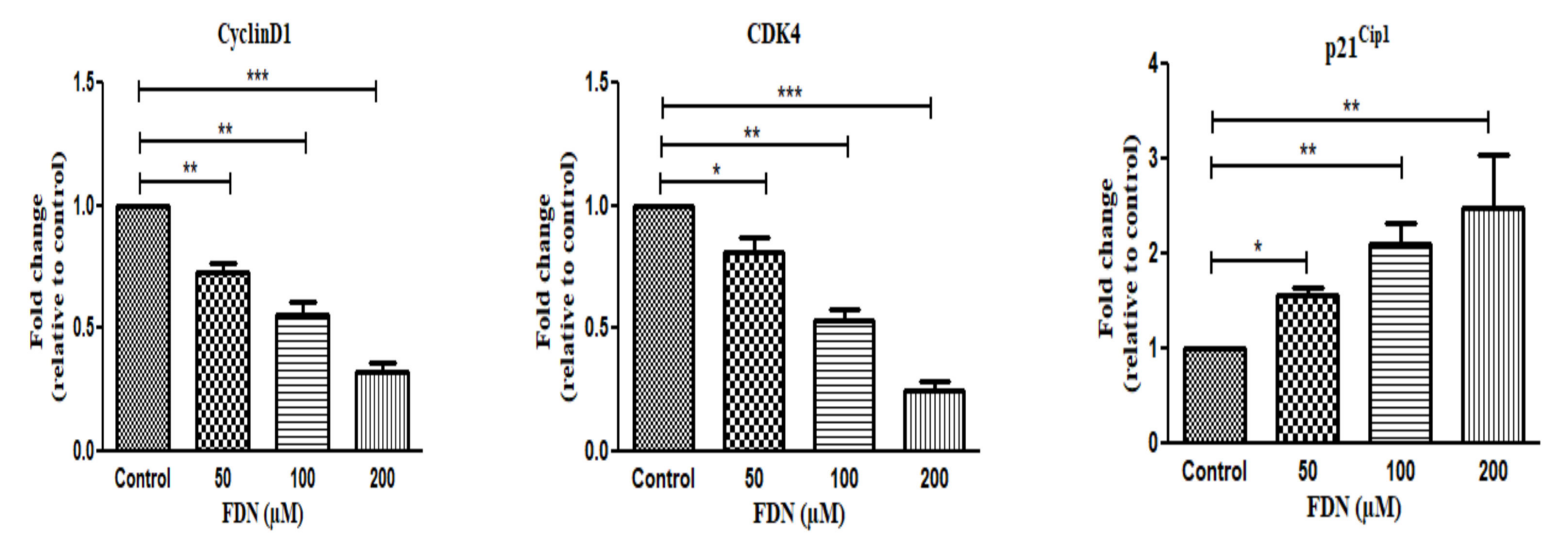
Effect of FDN on mRNA expression of genes involved in cell cycle progression. Fold change in mRNA expression level relative to control of *CyclinD1*, *CDK4*, and *p21Cip1* was analyzed quantitatively through RT-PCR. * p < 0.05, ** p < 0.01, *** p <0.001 vs. control.

Generally, the cell cycle is regulated in part by cyclins and their associated cyclin-dependent kinases (CDKs) [20]. Therefore, to gain further insight on the impact of FDN on cell cycle progression, the effect of FDN on cell cycle-associated genes, such as *cyclinD1*, *CDK4*, and *p21Cip1* genes, was investigated. As shown in Figure 8, FDN exposure resulted in a dose-dependent decline in the expression level of both *cyclinD1* and *CDK4* genes, which play a crucial role in cell progression through the G1 phase, compared to untreated cells. Treatment with FDN resulted in a significant decrease in cyclin D1 expression levels, respectively, compared to untreated cells. Similarly, treatment with 50, 100, and 200 µM of FDN triggered a dose-dependent reduction in *CDK4* expression levels, respectively, compared to untreated cells. On the other hand, FDN exposure substantially elevated the expression levels of the cell cycle inhibitor, *p21Cip1*, compared to untreated cells (Figure 8). Collectively, our results indicate that FDN could trigger cell cycle arrest via alleviating the expression levels of *cyclinD1* and *CDK4* genes, while elevating the expression of cell cycle inhibitor, *p21Cip1*.

### 3.6. Wnt/β-Catenin Signaling Cascade Regulates FDN-Induced Apoptosis in A549 Lung Cancer Cells

**Figure 9:**
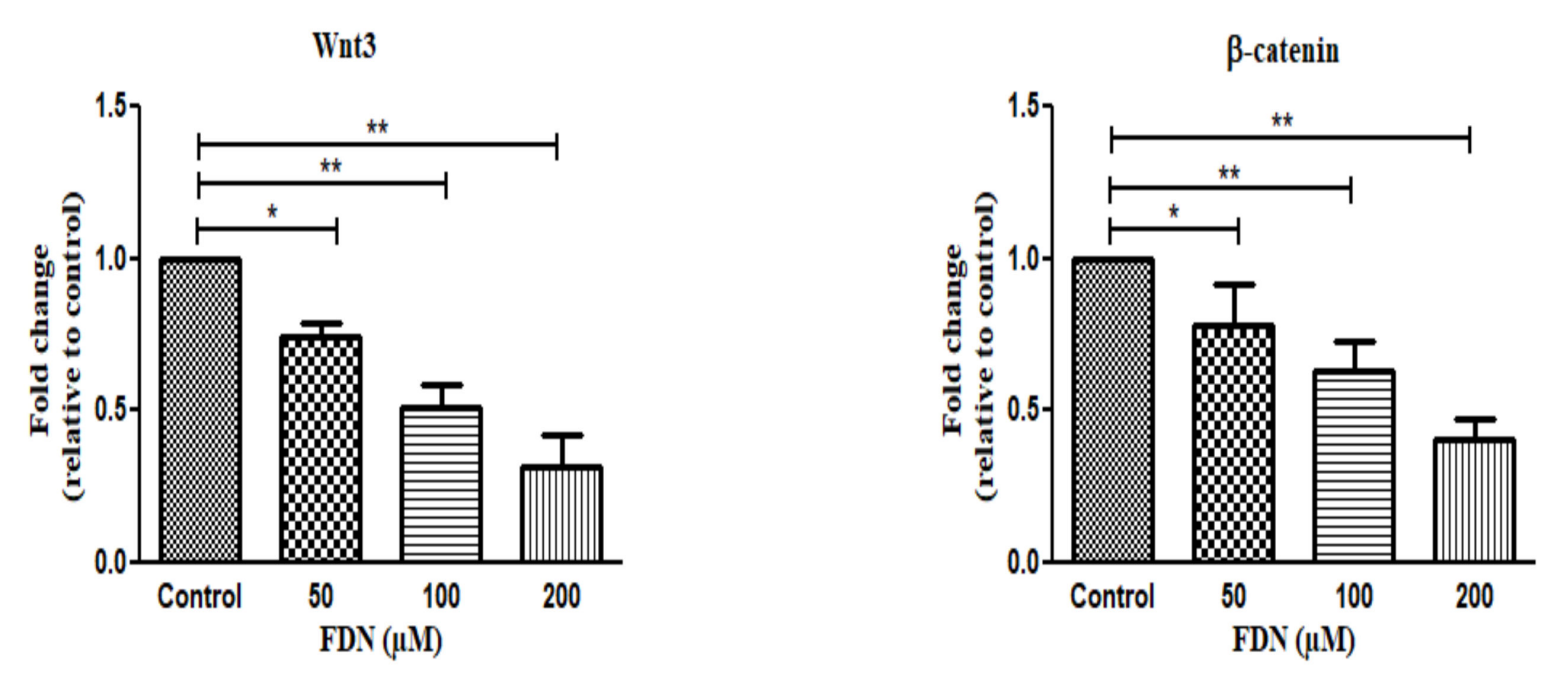
Effect of FDN on mRNA expression of genes involved in the Wnt signaling pathway. Fold change in mRNA expression level relative to the control of Wnt3 and β-catenin was analyzed quantitatively through RT-PCR. * p < 0.05, ** p < 0.01 vs. control.

Canonical Wnt/β-catenin signaling is activated by the nuclear translocation of β-catenin, culminating into transcriptional activation of downstream targets. Therefore, we explored the influence of FDN on the mRNA expression of some peculiar proteins representing this pathway. As shown in Figure 9, Wnt3 expression was substantially suppressed by treatment with FDN. The expression levels of Wnt3 in A549 cancer cells were 0.74, 0.51, and 0.31 upon treatment with 50, 100, and 200μm FDN, respectively, compared to untreated cells. Similarly, FDN inhibited β-catenin expression in a dose-dependent manner. The expression levels of β-catenin were significantly reduced to 0.78, 0.63, and 0.41 in both the nucleus and cytoplasm upon treatment with 50, 100, and 200μm FDN, respectively, compared to untreated cells. These findings show an FDN-mediated blockade of the Wnt/β-catenin cascade.

## 4. Discussion

Furanodienone (FDN) is an emerging bioactive furanosesquiterpenoid with diverse pharmacological activities, such as anti-inflammatory and antimicrobial activities [6]. Recently, many reports have emphasized its cytotoxic potential against different cancer cell lines, including colorectal cancer and breast cancer [7,9,21]. However, the antiproliferative potential of FDN against human lung cancer has not been elucidated yet. In the current study, we demonstrated, for the first time, that FDN could efficiently inhibit A549 cell proliferation (Figure 1). Furthermore, we declared the probable mechanism(s) underlying such potent cytotoxic effects against lung cancer cells. FDN induced G0/G1 cell cycle arrest in lung cancer cells (Figure 7). In addition, FDN efficiently triggered apoptosis within A549 cells via upregulating the expression of apoptotic proteins (Bax, Bad) and downregulating the expression of antiapoptotic proteins (Bcl-2) (Figure 6). Most importantly, FDN-treated A549 cells efficiently downregulated the Wnt signaling pathway (Figure 9), which was correlated with increased apoptosis as well as cell cycle arrest in A549 cells.

Anticancer effect is usually mediated by the inhibition of proliferation and cell cycle arrest. In this study, we demonstrated that FDN induces remarkable cell growth inhibition in A549 in a dose-dependent manner (Figure 1a), with maximum efficacy (>70% growth inhibition) observed at a concentration of 200 μM. Such a cytotoxic effect of FDN was comparable to that of a standard compound, cisplatin, signifying the efficiency of FDN against A549 NSCLC. Similar results were reported by Jiang et al. [7], who demonstrated that treatment of RKO and HT-29 colorectal cancer cells with 400 µM FDN for 24 h induced cell growth inhibition of ~50 and 40%, respectively. In the same context, Li et al. [21] revealed that FDN at a concentration of 160 μM could inhibit BT474 and SKBR3 breast cancer cell growth by only ~30 and 50%, respectively. Of interest, the calculated IC50, herein, for FDN against A549 cells was found to be 85.02 μM, which was much lower than that for FDN against RKO and HT-29 colorectal cancer cells (IC50 values were 156.4 and 251.1 μM, respectively) [7]. Collectively, these results emphasized the efficient anticancer potential of FDN against A549 lung cancer cells.

Furthermore, in order to demonstrate the antiproliferative efficacy of FDN, the distribution of treated cells in various cell cycle phases, namely the sub-G0/G1 (apoptosis), G0/G1, S, and G2/M phases, was detected using PI staining. Generally, G0/G1 and G2/M stages of the cell cycle are the major checkpoints and are implicated with critical roles in cell cycle regulation [22]. Our data reveal that FDN arrested the progression of cell cycle at G0/G1 in NSCLC A549 cells (Figure 7) by preventing the transition from G0/G1 to S, and thus resulting in their apoptosis. It is well documented that precise timing of cell-cycle transitions relies, at least in part, on the regulation of the activity of cyclins and their associated cyclin-dependent kinases (CDKs). Cyclin D1 plays a crucial role in regulating the transition from G1 phase of the cell cycle to S phase [23]. Furthermore, enhanced expression of cyclin D1 instigates profound proliferation of cells by altering the cell cycle′s homeostatic regulatory mechanisms and acts as a risk factor for the onset of cancers [24]. In the current study, treatment with FDN impeded the expression of cyclin D1 and CDK 4 and triggered the expression of the cell cycle inhibitor, *p21Cip1* (Figure 8), which collectively might account for the reduced cell viability and/or proliferation of A549 cells.

Apoptosis induction by drugs has emerged as an efficient therapeutic intervention against cancer cells. DNA fragmentation and chromatin condensation are the chief characteristics of apoptosis, and these were observed in NSCLC cells after treatment with FDN. Moreover, the results of the FITC-Annexin V/PI assay also confirmed the apoptotic efficacy of FDN in A549 cells. It was observed that FDN significantly increased the percentage of Annexin-V-positive cells in a dose-related manner in NSCLC A549 cells, indicating that FDN is an apoptotic agent.

The key role of mitochondria in the regulation of apoptosis is well acknowledged, making them an appealing target for the development of novel anticancer drugs [25]. In the current study, we revealed that FDN could induce mitochondrial membrane disturbance by dissipating the mitochondrial transmembrane potential (ΔΨm) (Figure 5). Furthermore, we demonstrated that FDN treatment triggered the upregulation of proapoptotic proteins (Bax and Bad), with mutual downregulation of antiapoptotic (Bcl-2) proteins in A549 cells. The Bcl-2 protein family regulates cell death largely by direct binding interactions that govern mitochondrial outer membrane permeabilization, which eventually results in the irreversible release of intermembrane space proteins, caspase activation, and apoptosis. Collectively, these results imply that mitochondria are at the nexus of sensing and processing the stress induced by FDN in lung cancer cells.

Caspases are a family of cysteine proteases that play important roles in regulating apoptosis [26]. Accordingly, to gain further insight into the mechanisms involved in the induction of apoptosis by FDN, caspase activities were evaluated. Our results revealed that FDN enhanced the activity of not only the initiator caspase-9, but the effector caspase-3 as well in a dose-dependent manner (Figure 2). Interestingly, the caspase inhibitors, Z-LEHD-FMK (caspase-9 inhibitor) and Z-VAD-FMK (caspase-3 inhibitor), completely inhibited the apoptogenic activity of FDN (Figure 3). These results suggest that caspase-dependent processes are involved in the apoptogenic activity of FDN.

**Scheme 2:**
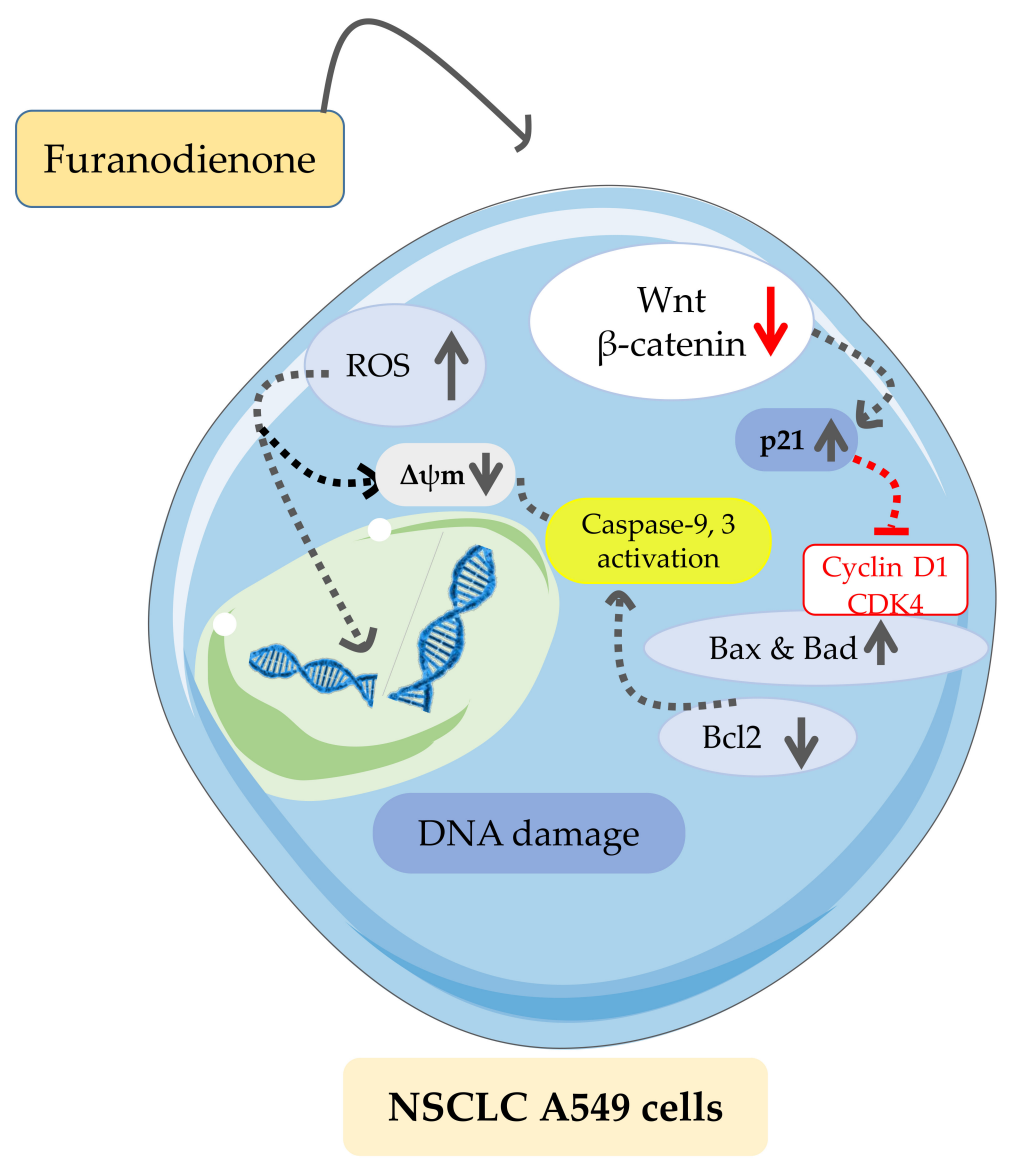
Cartoon depicting the plausible mechanism of the cytotoxic effect of FDN against NSCLC A549.

The canonical Wnt/β-catenin signaling cascade is widely known to be functionally important in the regulation of a myriad of biological processes, including cell proliferation, differentiation, and apoptosis. Modulations in this pathway are involved in the pathogenesis of various types of carcinomas [26,27,28,29]. Alterations within the Wnt/β-catenin are prominent in various human carcinomas. The Wnt signaling cascade is important in NSCLC cell lines. Wnt-1, -2, -3, and -5a components of the Wnt pathway are found to be overexpressed along with the frizzled-8, disheveled, porcupine, and TCF-4 in NSCLC, which is associated with poor prognosis [29]. In addition, the oncogenic hyper-activated Wnt/β-catenin is prominent in lung cancer, playing an important role in tumorigenesis, prognosis, and resistance to therapy [30]. Currently, many researches are focusing on the development of specific inhibitors of WNT/β-catenin signaling for cancer therapies [31,32]. Herein, it was obvious that Wnt3 and β-catenin expression levels were reduced in A549 cells post-treatment with FDN in a dose-dependent manner (Figure 9). These results suggest that FDN exerted its anticancer effect, presumably via downregulating the Wnt/β-catenin signaling cascade in A549 cells. Nevertheless, it is important to underscore that it is difficult to target only the WNT pathway without interfering with other signaling pathways. Recent reports have revealed the extensive crosstalk between Wnt/β-catenin and mitogen-activated protein kinase (MAPK) signaling in cancer [33,34]. Hu and his colleagues [7] have reported that FDN could induce G0/G1 arrest and trigger cell death via the ROS/MAPKs-mediated caspase-dependent pathway in human colorectal cancer cells. Furthermore, Jeong et al. [35] have demonstrated that, in colorectal cancer, Wnt/β-catenin signaling pathway stimulation efficiently triggered the activation of the MAPK pathway through Ras stabilization, indicating a positive crosstalk. Collectively, in this study, FDN might induce apoptosis and cell cycle arrest in A549 lung cancer cells via a crosstalk between Wnt/β-catenin and MAPK signaling (Scheme 2); however, further investigations are required to provide a better understanding of FDN-mediated anticancer effect on human adenocarcinomas.

## 5. Conclusions

In this study, we demonstrated the efficacy of the natural compound furanodienone (FDN) to induce apoptosis in non-small cell lung cancer. Our findings not only provide the first evidence of FDN potential as a novel therapeutic option for the treatment of lung cancer, but also provide insight into the molecular mechanism(s) by which FDN induces cell death. FDN suppressed the proliferation of A549 lung cancer cells by restraining the progression of the cell cycle at G0/G1 phase, and caused cell apoptosis, at least in part, via downregulation of the canonical Wnt signaling pathway. Collectively, the natural compound, FDN, might represent a promising adjunctive drug with low toxicity targeting on lung cancer. However, in vivo model-based exploratory studies elucidating the mechanistic details of FDN are still a prerequisite to fully validate the anticancer potential of FDN against NSCLC.

## Figures and Tables

**Table 1:** Primer sequences used in the study.

Genes	Forward Sequence	Reverse Sequence
*GAPDH*	CGACCACTTTGTCAAGCTCA	CCCCTCTTCAAGGGGTCTAC
*Bax*	GCCCTTTTGCTTCAGGGTTT	TCCAATGTCCAGCCCATGAT
*Bad*	CCTCAGGCCTATGCAAAAAG	AAACCCAAAACTTCCGATGG
*Bcl2*	GATTGTGGCCTTCTTTGAG	CAAACTGAGCAGAGTCTTC
*CyclinD1*	CCGTCCATGCGGAAGATC	GAAGACCTCCTCCTCGCACT
*CDK4*	CCTGGCCAGAATCTACAGCTA	ACATCTCGAGGCCAGTCATC
*p21^Cip1^*	TCCAGGTTCAACCCACAGCTACTT	TCAGATGACTCTGGGAAACGCCAA
*Wnt3*	CGCTCAGCTATGAACAAGCA	AAAGTTGGGGGAGTTCTCGT
*β-catenin*	TCTGAGGACAAGCCACAAGATTACA	TGGGCACCAATATCAAGTCCAA

## Data Availability

The data presented in this study are available on request from the corresponding author.

## Supplementary Materials

The following are available online at https://www.mdpi.com/article/10.3390/life12010114/s1, Figure S1: Cell viability of A549 cells treated with varying concentrations of FDN.
