# Supplementary figures and images for: Screening of Apoptosis Pathway-Mediated Anti-Proliferative Activity of the Phytochemical Compound Furanodienone against Human Non-Small Lung Cancer A-549 Cells
Source: Life (Basel). 2022 Jan 13;12(1):114. doi: 10.3390/life12010114 (PMC8779876; doi:10.3390/life12010114)

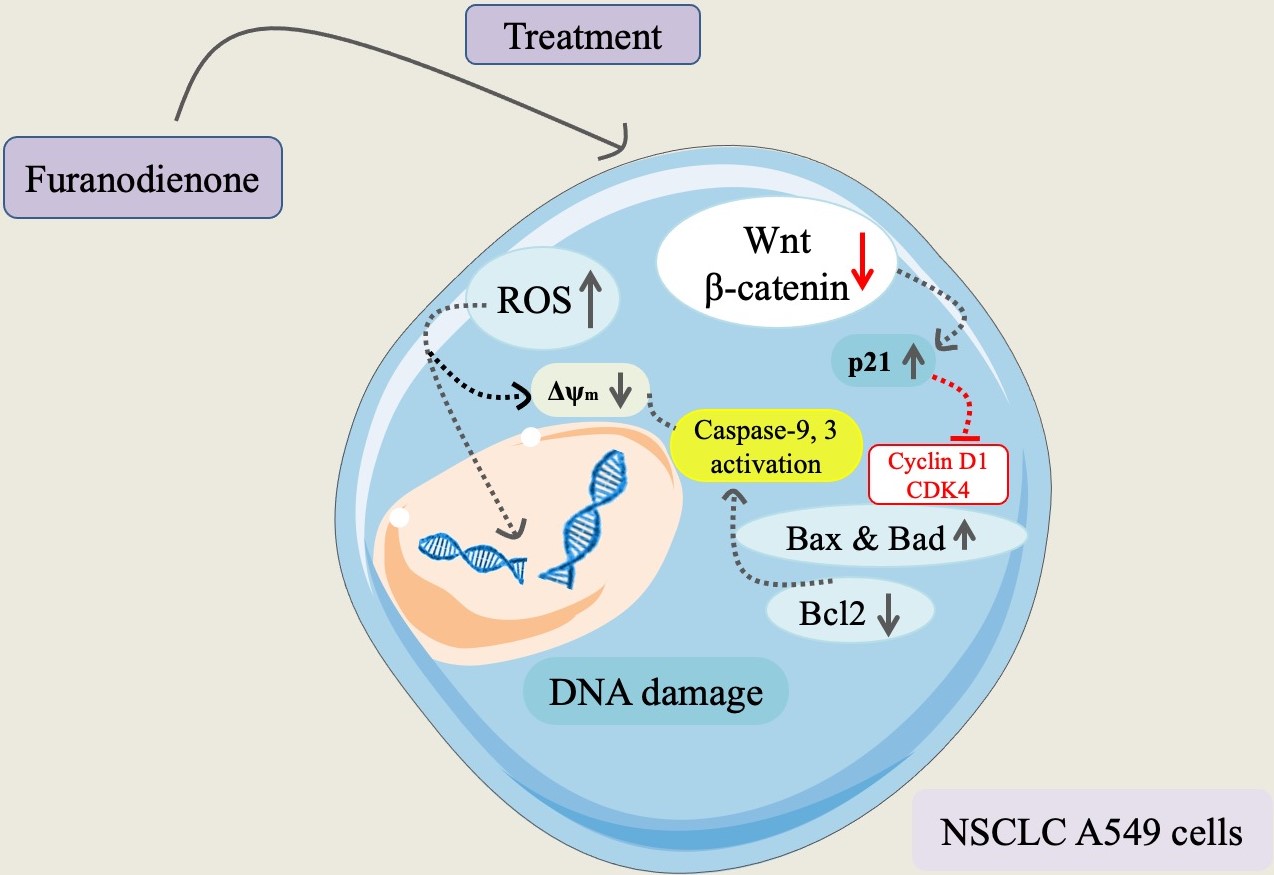

Supplement: Supplementary file 1 [file life-12-00114-s001.zip › life-1518005 supp. Figure S1.jpg]
